# DNA binding specificity of ATAF2, a NAC domain transcription factor targeted for degradation by Tobacco mosaic virus

**DOI:** 10.1186/1471-2229-12-157

**Published:** 2012-08-31

**Authors:** Xiao Wang, James N Culver

**Affiliations:** 1 Institute for Bioscience and Biotechnology Research, University of Maryland, College Park, MD, 20742, USA; 2 Department of Plant Sciences and Landscape Architecture, University of Maryland, College Park, MD, 20742, USA

**Keywords:** Transcriptional reprogramming, NAC binding, *Cis*-regulatory sequence

## Abstract

**Background:**

Control of the host transcriptome represents a key battleground in the interaction of plants and pathogens. Specifically, plants have evolved complex defense systems that induce profound transcriptional changes in response to pathogen attack while pathogens have evolved mechanisms to subvert or disable these defenses. Several NAC transcription factors such as ATAF2 have been linked to plant defense responses, including those targeting viruses. The replication protein of *Tobacco mosaic virus* (TMV) has been shown to interact with and target the degradation of ATAF2. These findings suggest that the transcriptional targets of ATAF2 are involved in defense against TMV.

**Results:**

To detect potential ATAF2 transcriptional targets, a genomic pull-down assay was utilized to identify ATAF2 promoter binding sequences. Subsequent mobility shift and DNA footprinting assays identified a 30-bp ATAF2 binding sequence. An *in vivo* GUS reporter system confirmed the function of the identified 30-bp binding sequence as an ATAF2 specific transcriptional activator *in planta*. Gel filtration studies of purified ATAF2 protein and mutagenesis studies of the 30-bp binding sequence indicate ATAF2 functions as a dimer. Computational analysis of interacting promoter sequences identified a corresponding 25-bp A/T-rich consensus sequence with repeating [GC]AAA motifs. Upon ATAF2 induction real-time qRT-PCR studies confirmed the accumulation of select gene transcripts whose promoters contain this consensus sequence.

**Conclusion:**

We report the identification of a *cis*-regulatory binding sequence for ATAF2. Different from other known NAC protein binding sequences, the A/T-rich ATAF2 binding motif represents a novel binding sequence for NAC family proteins. Combined this information represents a unique tool for the identification of ATAF2 target genes.

## Background

Plants have evolved sophisticated sensing systems that utilize a multitude of components to translate the perception of a pathogen into the induction of defense responses. In particular, alterations in gene expression as directed by defense-associated transcription factors (TFs) such as ERF, NAC, WRKY, and bZip represent important host responses that occur during pathogen attack [1-3]. In contrast, reprograming gene expression is an important strategy pathogens use to disable host defenses and enhance their ability to establish an infection. To counter the induction of these defenses pathogens have evolved mechanisms to override host transcriptional responses either through the targeted disruption of defense associated TFs or through the production of their own factors for controlling transcription [4,5]. Characterization of pathogen targeted TFs and the regulatory networks they control are thus essential to developing a full understanding of plant defense responses.

Previously, we reported that the Arabidopsis TF ATAF2 (At5g08790) is induced in response to a *Tobacco mosaic virus* (TMV) infection and that TMV subsequently targets ATAF2 for degradation through an interaction with the viral 126 kDa replication protein [6] . ATAF2 is a member of the NAC (NAM, ATAF1/2, CUC2) family of plant specific TFs and is induced in response to tissue wounding and pathogen infection [6,7]. We also observed that overexpression of ATAF2 resulted in the induction of salicylic acid (SA) mediated defense associated marker genes *PR1* and *PR2*, conversely these genes had reduced transcript levels in ATAF2 knockout or repressor lines [6]. Furthermore, ATAF2 overexpression inhibited TMV accumulation in inoculated tissues. These findings suggest that ATAF2 plays a role in the regulation of host basal defense responses and that TMV targets ATAF2 for degradation as a means to disrupt these defense pathways.

NAC domain TFs such as ATAF2 make up a large plant specific family of proteins with ~105 NAC genes in Arabidopsis and ~ 75 in rice [8]. All members within this family contain a highly conserved N-terminal NAC domain and a divergent C-terminal transcription activation region (TAR). NAC genes have been widely reported to be involved in plant morphogenesis/organ development, senescence and abiotic/biotic stresses [9-13]. In addition, several NAC proteins are reported to interact with viral proteins. These include interactions between the NAC containing GRAB proteins and the Geminivirus RepA protein [14] and the Arabidopsis TIP protein with the *Turnip crinkle virus* coat protein [15]. These interactions are implicated in the modulation of virus replication and the induction of host defense responses. Combined these findings suggest that NAC domain proteins are key TFs controlling molecular pathways that are of importance to virus biology.

To further understand the role of ATAF2 in virus biology we utilized a genomic pull-down assay to identify potential ATAF2 target sequences from the Arabidopsis genome. An analysis of the DNA sequences bound by ATAF2 led to the identification of a 25-bp ATAF2 specific consensus binding sequence. This binding sequence is sufficient to promote ATAF2 mediated gene transcription and is unique in comparison to previously reported NAC protein binding domains [16,17].

## Results

### Identification of ATAF2 binding sequences

**Figure 1:**
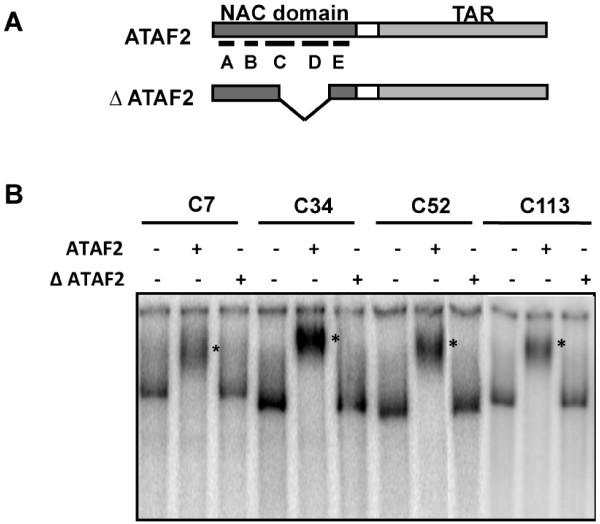
**ATAF2 binds DNA sequences from the genomic pull-down assay.** (**A**) Schematic representation showing ATAF2 and **∆**ATAF2 deletion constructs. NAC domains C and D cover the DNA binding domain. (**B**) EMSA assay confirming ATAF2 and not **∆**ATAF2 binds to DNA clones identified in genomic pull-down assays. DNA probes were prepared by PCR-amplification followed by end-labeling with [γ-^32^P]ATP. The asterisk indicates the shifted band after ATAF2 binding. The four clones tested (C7, C34, C52, and C113) were all located within 1000 bp upstream of the coding sequences of At1G08540, At1G68907, At3G26540, and At3G11700 respectively.

ATAF2 binding sequences were identified via an immuno-pull-down assay using purified hexa-histidine tagged ATAF2 and genomic DNA isolated from *Arabidopsis thaliana* ecotype Shahdara. In addition, a hexa-histidine tagged ATAF2 deletion construct, ΔATAF2, lacking the putative DNA binding subdomains C and D, nucleotides 172 – 377, was used as a negative control (Figure 1A). In this assay the hexa-histidine tagged ATAF2 or ΔATAF2 proteins were mixed with *EcoR*I and *Taq*I cut Shahdara genomic DNA and precipitated using anti-PolyHis antibody [18]. Both ATAF2 and ΔATAF2 bound DNA fragments were subsequently PCR-amplified and cloned into a plasmid vector. The number of DNA clones derived from ATAF2 bound complexes (~600 clones) were 50-fold higher than that derived from ΔATAF2 bound complexes (12 clones), reflecting the deletion of the putative DNA binding regions. Of 97 ATAF2 clones randomly selected for sequencing 47 were located upstream and within 3000 basepairs (bp) of a known or predicted genomic translational start site (Table 1).

**Table 1:** Putative ATAF2 target genes identified via a genomic pull-down assay

**Clone #**	**AGI**	**Annotation**
C7*	AT1G08540	RNA Polymerase sigma subunit
C9	AT3G61111	Structural constituent of ribosome
C18*	AT5G61810	Pentatricopeptide (PPR) repeat-containing protein
C20	AT5G39460	F-box family protein
C22	AT2G38940	AtPT2, phosphate transporter 2
C23	AT4G24660	Homeobox protein 22, AtHB22
C25	AT3G44470	Transposable element gene
C28*	AT4G07840	Transposable element gene
C30*	AT5G24350	Unknown protein
C32*	AT2G28390	SAND family protein
C33	AT2G02840	Unknown protein
C34*	AT1G68907	Defensin-like (DEFL) family protein
C37	AT4G05150	Octicosapeptide/Phox/Bem1p (PB1) domain-containing protein
C38	AT1G26620	Unknown protein
C39*	AT1G08370	DCP1 involved in mRNA decapping
C41	AT1G59820	AMINOPHOSPHOLIPID ATPASE3
C42	AT1G70170	Matrix metalloproteinase, MMP
C45*	AT3G19080	SWIB complex BAF60b domain-containing protein
C49	AT1G55060	Ubiquitin-like gene
C52*	AT3G26540	Pentatricopeptide (PPR) repeat-containing protein
C56	AT3G57220	UDP-GlcNAc:dolichol phosphate N-acetylglucosamine-1-phosphate transferase
C58	AT1G01210	DNA-directed RNA polymerase III family protein
C59	AT5G21482	Cytpkinin oxidase
C64	AT1G14180	Protein binding / zinc ion binding
C65*	AT3G01880	Unknown protein
C66*	AT5G27902	Transposable element gene
C81	AT2G37160	Transducin family protein / WD-40 repeat protein
C83*	AT4G13440	Calcium-binding EF hand family protein
C85	AT3G22300	Nuclear-encoded gene for mitochondrial ribosomal small subunit protein S10
C87	AT4G09584	Unknown pseudogene
C92	AT3G53365	Unknown gene
C96	AT5G13190	Unknown protein
C98	AT3G56600	Inositol or phosphatidylinositol kinase/ phosphotransferase
C100*	AT1G70070	EMB25, Embryo defective 25
C104*	AT3G62060	Pectinacetylesterase family protein
C107	AT4G01533	Unknown gene
C108*	AT4G19570	DNAJ heat shock N-terminal domain-containing protein
C109	AT3G10912	CPUORF63
C110*	AT4G22980	Unknown protein
C111	AT2G12230	Pseudogene, C-1-tetrahydrofolate synthase
C113*	AT3G11700	FASCICLIN-LIKE ARABINOGALACTAN PROTEIN 18 PRECURSOR, FLA18
C115	AT4G20010	Plastid transcriptionally active 9 (PTAC9)
C116*	AT5G56550	Oxidative stress 3 (OXS3)
C120*	AT1G24070	Transposable element gene
C121	AT3G33100	Transposable element gene
C123*	At3G59050	Polyamine oxidase 3 (ATPAO3)
C131*	AT1G33010	F-box family protein

The identified ATAF2 interacting sequences were upstream and within 3000 bp of a translation start codon. Those marked by an * are upstream and within 1000 bp of a start codon.

To confirm that ATAF2 binds to the immunoprecipitated genomic DNA fragments, a representative group of four clones covering sequences within 1000 bp of the translational start sites for At1g08540, At1g68907, At3g26540, and At1g01210 were selected for electrophoretic mobility shift assay (EMSA) using purified recombinant hexa-histidine tagged ATAF2. We speculated that proximity to a translational start site would enhance the likelihood that these sequences function in ATAF2-mediated gene regulation. DNA fragments from the four selected clones were prepared by PCR amplification and P^32^ end-labeled. Gel shift assays for all tested clones produced a mobility shift in the presence of purified ATAF2 protein but not in the presence of purified ΔATAF2 protein (Figure 1B).

### Identification of a 30-bp ATAF2 binding sequence

**Figure 2:**
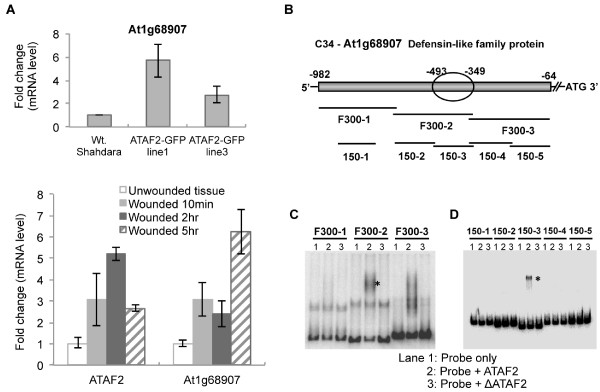
**ATAF2 induces the transcription and binds to a 150-bp fragment within the promoter region of a defensin-like family protein (At1g68907).** (**A**) Real-time qRT-PCR showing enhanced expression of At1g68907 in two 35S::ATAF2-GFP overexpression lines and in wounded Arabidopsis Shahdara plant tissue. RNA was extracted from five independent test plants. The data represents the average ± standard deviation from duplicate qRT-PCR reactions. (**B**) Schematic representation of the At1g68907 promoter fragment investigated for ATAF2 binding activity. EMSA assay showing ATAF2 binds to the DNA fragments F300-2 (**C**) and 150–3 (**D**) in the promoter region of At1G68907. The asterisk indicates the shifted band after ATAF2 binding.

One clone showing a strong mobility shift in the presence of ATAF2, designated C34, was selected for further studies aimed at identifying the specific DNA sequences targeted by ATAF2. The C34 DNA clone is located upstream (−64 nt to −982 nt) of the coding region for the Arabidopsis defensin-like protein At1g68907. To determine the likelihood that the defensin-like gene was regulated by ATAF2 and thus a good candidate gene for further analysis, we examined its expression levels in two independent Arabidopsis ecotype Shahdara ATAF2 overexpression lines as well as in response to tissue wounding (Figure 2A) [6]. Quantitative RT-PCR analysis showed At1g68907 was transcriptionally induced by an average ~4-fold in the two ATAF2 overexpression lines as well as in wounded Shahdara leaf tissues where expression of ATAF2 is also induced (Figure 2A). Transcriptional induction of At1g68907 thus corresponds with ATAF2 expression, either by transgene overexpression or wound induction of the endogenous gene, consistent with a role for ATAF2 in the regulation of this gene.

To narrow down the ATAF2 binding sequence the C34 fragment was sub-divided into three segments and each fragment was examined by EMSA for ATAF2 binding (Figure 2B). The second 300-bp fragment, F300-2, showed significant binding activity while the third fragment F300-3 showed relatively weak binding activity and the first fragment, F300-1, displayed no binding activity (Figure 2C). None of the fragments bound to ΔATAF2 (Figure 2C). Fragments, F300-2 and F300-3 were further subdivided into four ~150-bp fragments. A 150-bp fragment, 150–1, from F300-1, which showed no ATAF2 binding activity, was used as a negative control. Results indicated that fragment 150–3, covering nt −493 to −349 from the original 918-bp C34 clone was responsible for the observed ATAF2 binding (Figure 2D).

**Figure 3:**
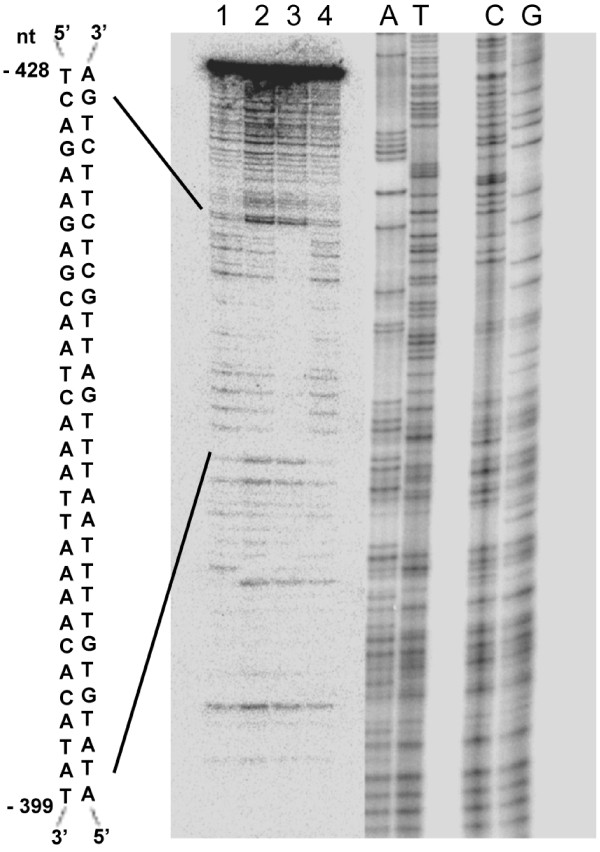
**DNase I footprinting analysis showing ATAF2 binds and protects a 30-bp sequence within the 150–3 promoter fragment of At1G68907.** The sequence of the protected region is shown on the left. Lane 1, no protein added; lane 2, 5 mM ATAF2 added; lane 3, 25 mM ATAF2 added; lane 4, 25 mM ΔATAF2 added. Sequencing ladders (A, T, C, G) were run in adjacent lanes to provide a positional reference.

DNase I footprinting was used to identify the specific region of the interacting 150–3 C34 fragment protected by ATAF2 binding. Results demonstrated that only one region was substantially protected from DNase I digestion (Figure 3). Sequence analysis of this region revealed it to be a 30-bp sequence (nt −428, TCAGAAGAGCAATCAAATTAAAACACATAT, nt −399). This protected region likely represents an ATAF2 *cis*-regulatory element.

### ATAF2 30-bp binding sequence functions in transcriptional activation

**Figure 4:**
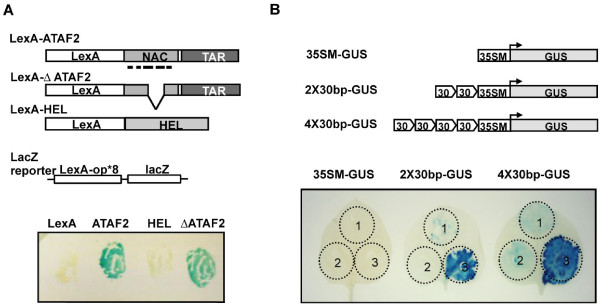
**ATAF2 functions as a transcriptional activator.** (**A**) β-galactosidase (LacZ) assay indicating both ATAF2 and ΔATAF2 are capable of activating *lacZ* expression in yeast. L40 yeast expressing an integrated Lac-Z reporter were transformed with constructs LexA (empty), LexA-ATAF2, LexA-HEL, or LexA-ΔATAF2. HEL represents TMV encoded helicase domain and was used as a negative control. (**B**) ATAF2, but not ΔATAF2, trans-activates *GUS* expression via the 30 bp ATAF2 binding sequence. The reporter constructs 35SM-GUS, 2X30bp-GUS, or 4X30bp-GUS were co-expressed with the pBin empty vector (circle 1), pBin/ΔATAF2 (circle 2) or pBin/ATAF2 (circle 3) in *N. benthamiana* leaves.

To determine if the identified 30-bp segment was sufficient to promote *in vivo* transcriptional activation by ATAF2, we first used a yeast lacZ reporter system to determine if the ATAF2 and ΔATAF2 constructs, both of which contain the putative transcriptional activation region, can function as transcriptional activators in yeast when fused to the LexA DNA-binding domain (Figure 4A). Results indicated that when fused to the LexA DNA binding domain both ATAF2 and ΔATAF2 proteins function in the transcriptional activation of the *LacZ* open reading frame (Figure 4A). Thus, given the presence of a DNA specific binding domain both ATAF2 constructs can function as transcriptional activators.

To confirm the *in planta* function of the identified 30-bp *cis*-regulatory element in ATAF2-mediated gene expression, reporter constructs containing either two (2X) or four (4X) tandem repeats of the 30-bp sequence were engineered upstream (−49 nt) of the minimal 35S CaMV promoter and investigated for the ability to drive β-glucuronidase (GUS) transcription (Figure 4B). The resulting GUS reporter constructs were agroinfiltrated into leaves of *Nicotiana benthamiana* in combination with 35S agro-expression constructs for ATAF2, ΔATAF2 or an empty cassette vector. Results revealed little GUS activity in plant tissues co-infiltrated with either the 2X or 4X repeat constructs and the empty cassette vector (Figure 4B). However, when the ATAF2 expression vector was co-infiltrated with either the 2X or 4X repeat constructs, GUS activity dramatically increased (Figure 4B). In contrast, the 35S minimal promoter construct yielded little GUS activity when co-expressed with the ATAF2 expression vector. Furthermore, the co-expression of 2X or 4X repeat constructs with the ΔATAF2 construct, which lacks the putative ATAF2 DNA binding domain, also failed to induce significant GUS activity (Figure 4B). Combined these results indicate that ATAF2 can utilize the identified 30-bp regulatory binding element *in vivo* for transcriptional activation.

### TMV infection increases GUS activity driven by the 30-bp ATAF2 binding element

**Figure 5:**
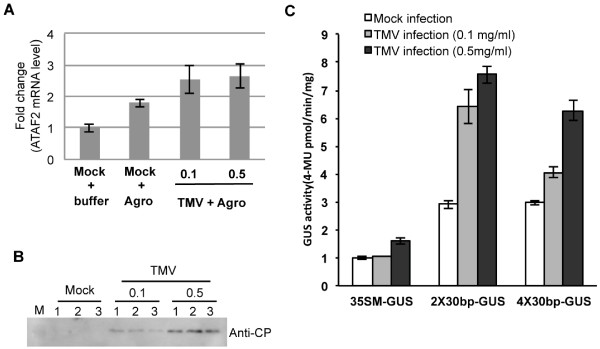
**TMV infection enhances GUS activity driven by the ATAF2 binding sequence.** (**A**) Real-time qRT-PCR analysis showing increased ATAF2 expression in Agro-infiltrated and TMV-inoculated tissues at 6 dpi. Numbers 0.1 and 0.5 are the mg/ml of virus used for inoculation. RNA was extracted from three independent test plants. Data represents average ± standard deviation from triplicate qRT-PCR reactions. (**B**) TMV coat protein specific Western-blot showing the level of virus accumulation in three independent samples of TMV-inoculated tissues. (**C**) Enhanced GUS activity observed in TMV-inoculated tissues. At four dpi TMV-infected Arabidopsis plants were agro-infiltrated with the 35S minimal promoter (35SM-GUS), the 2X30bp-GUS, or the 4X30bp-GUS reporter constructs. At six dpi tissue punches from three independent leaves were collected and tested by fluorometric assay for GUS activity. Data represents the average ± standard deviation.

Previously, we demonstrated the transcriptional induction of ATAF2 within TMV inoculated leaf tissues [6]. To determine whether the identified 30-bp binding sequence functions in response to ATAF2 produced endogenously during an infection, TMV inoculated Arabidopsis leaf tissues were agro-infiltrated with either the 2X or 4X GUS reporter constructs at 4 dpi. GUS activity was quantified two days post agro-infiltration. Both the levels of ATAF2 mRNA as well as GUS activity increased within agro-infiltrated mock-inoculated tissues, indicating that agro-infiltration alone is sufficient to induce ATAF2 (Figure 5A). However, within TMV infected tissues, both 2X and 4X constructs containing the 30-bp regulatory sequence displayed significantly greater increases in GUS activity (averaging ~2-fold increases across treatments) than observed in mock-inoculated tissues or tissues infiltrated with the 35S minimal promoter construct (Figure 5C). This result corresponds with the higher levels of ATAF2 mRNA detected in the TMV infected leaves (Figure 5A and B). These findings are consistent with the induction of ATAF2 in response to TMV infection and indicates that the 30-bp binding element is a functional target of endogenously expressed ATAF2.

### Mutational analysis of the ATAF2 binding element

**Figure 6:**
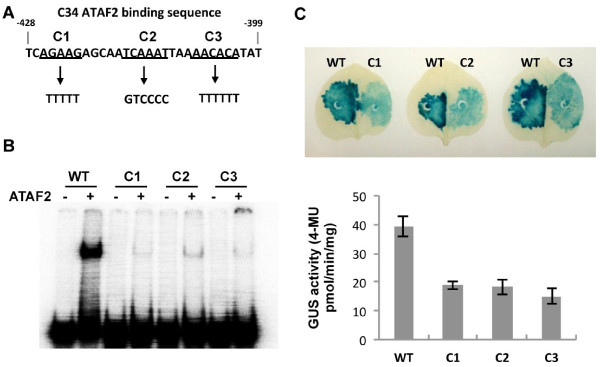
**Mutations within the 30-bp sequence affect ATAF2-DNA binding activity.** (**A**) Schematic diagram of mutants created within the 30-bp ATAF2 binding sequence. (**B**) EMSA assay showing all three mutations have reduced ATAF2 binding activity. (**C**) Both GUS staining assay and GUS quantification assay confirming that all three mutants affected ATAF2-DNA binding activity *in vivo*. Tissues punches from three independent leaves were collected at two days post agro-infiltraton and tested by fluorometric assay for GUS activity. Data represents the average ± standard deviation.

To analyze the identified ATAF2 binding element, three substitutions replacing 5 to 6 nucleotides within the 30-bp binding sequence were created and tested both *in vitro* and *in vivo* (Figure 6A). Substitutions were designed to alter segments of the binding element to determine if any specific region played a greater role in ATAF2 binding. The first mutation covers nts 3–7, replacing AGAAG with TTTTT (designated as C1). The second mutant covers nts 13–18, substituting TCAAAT to GTCCCC (designated as C2). The third mutant covers nts 22–27, exchanging AACACA to TTTTTT (designated as C3). EMSA results showed that all three substitutions displayed substantial reductions of >85% in ATAF2 binding, suggesting that the entire sequence is required for ATAF2 binding (Figure 6B).

ATAF2 binding elements carrying the C1, C2, and C3 were also introduced into the Agrobacterium 2X-GUS reporter construct and tested for GUS activity when co-expressed with ATAF2 in *N. benthamiana* leaves. In these assays, all three substitutions displayed >50% reductions in the levels of GUS activity when compared to the unmodified binding element 2X-GUS construct (Figure 6C). This finding is consistent with the EMSA analysis and indicates that the entire binding element is required for full activity.

**Figure 7:**
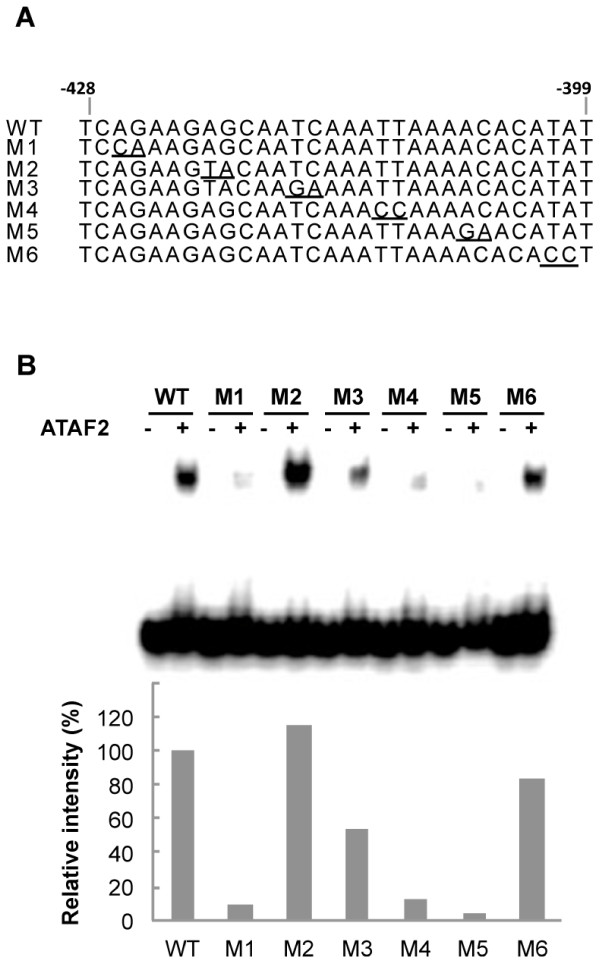
**Scanning mutagenesis analysis of the ATAF2 binding sequence.** (**A**) Six different two base substitutions (M1 – M6) were created within the 30-bp binding sequence. Specific mutations are underlined. (**B**) EMSA assays testing ATAF2-binding activity. The binding activity was expressed relative to ATAF2 binding to wild-type 30-bp sequence as 100%.

To further investigate the ATAF2 binding sequence a series of six two-base substitutions within the identified 30-bp DNA fragment were created and tested for their effects on ATAF2 binding (Figure 7A). From gel shift assays, mutations M1 (base substitutions from AG to CA at nt position 3 and 4), M4 (TT to CC at nt position 18 and 19), and M5 (AC to GA at nt position 23 and 24) showed the most dramatic reduction in ATAF2 binding activities of 91%, 87%, and 96%, respectively (Figure 7A & B). Mutation M3 (TC to GA at nt position 13 and 14) showed a partial reduction of 47% (Figure 7B). The other two mutations M2 and M6 did not dramatically reduce the binding of ATAF2 to the identified 30 bp element (Figure 7B). These findings further indicate that sequences covering nearly the entire 30-bp binding domain contribute to ATAF2 binding.

### Dimerization of the ATAF2 NAC domain

**Figure 8:**
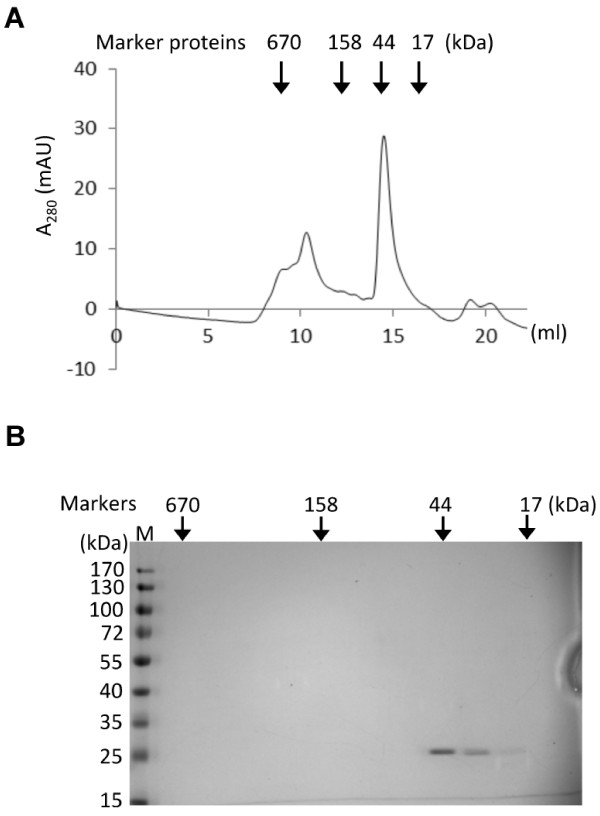
**The ATAF2 NAC domain forms a dimer.** (**A**) Sephadex-200 size-exclusion chromatography of the purified ATAF2 NAC domain. Molecular markers include thyroglobulin (670 kDa), gamma globulin (158 kDa), ovalbumin (44 kDa), and myoglobin (17 kDa). (**B**) Fractions from the size-exclusion column were analyzed by SDS-PAGE and stained with Coomassie blue to confirm the presence of the ATAF2 NAC domain within the dimer fractions.

Having demonstrated that nearly the entire 30-bp sequence is required for optimal ATAF2 binding, we speculated that ATAF2 may function as a dimer or multimer, similar to other reported NAC domain proteins [19,20]. To characterize the quaternary structure of ATAF2, the ATAF2 DNA-binding domain (NAC domain) was expressed from bacteria and the purified protein examined using size-exclusion chromatography. Results indicate that the purified ATAF2 NAC domain peptide eluted as a protein of around ~44 kDa, which is twice the molecular weight of the monomeric NAC domain (22.8 kDa) (Figure 8A & B), indicating that the DNA binding domain of ATAF2 forms a dimer.

### ATAF2-mediated regulation of selected pull-down sequences

To examine the ability of ATAF2 to alter the transcription of genes identified in the pull down assay, a group of nine genes were selected and examined by real-time qRT-PCR for expression in both wounded wild-type and wounded ATAF2 knockout (KO) line (SALK_136355) leaf tissues. Wounding was used to induce endogenous ATAF2 expression with tissues harvested after 5 hours [6,7]. Genes were selected based on the location of an ATAF2 binding sequence within 1000 bp of a translational start codon. In addition, defensin-like protein At1g68907, corresponding to clone C34, was included in the group as a control. Consistent with our previous results the transcript of At1g68907 showed a reduced accumulation in wounded tissues from the ATAF2 KO line (Table 2). Furthermore, 7 of the 8 additional transcripts examined showed at least a two-fold reduction in accumulation within wounded tissues of the ATAF2 KO line (Table 2). These findings indicate that a significant portion of the genes whose promoter sequences were pulled-downed by ATAF2 are directly affected by its presence and are thus likely to carry a functional ATAF2 binding sequence.

**Table 2:** Gene expression analysis on ATAF2 candidate target genes

**Clone number**	**AGI**	**Annotation**	Wt Col. 5 hr wounded vs. non-wounded*	ATAF2 KO-136355 5 hr wounded vs. non-wounded*
	AT5G08790	ATAF2	5.37	1
C7	AT1G08540	RNA Polymerase sigma subunit	1.42	0.47
C32	AT2G28390	SAND family protein	1.64	0.91
C34	AT1G68907	Defensin-like (DEFL) family protein	3.63	0.99
C52	AT3G26540	Pentatricopeptide (PPR) repeat-containing protein	2.00	0.78
C104	AT3G62060	Pectinacetylesterase family protein	2.91	1.06
C108	AT4G19570	DNAJ heat shock N-terminal domain-containing protein	8.06	2.17
C113	AT3G11700	Fasciclin-like arabinogalactan protein 18 precursor, FLA18	3.69	1.02
C116	AT5G56550	Oxidative stress 3, OXS3	3.68	1.55
C123	AT3G59050	Polyamine oxidase 3, AtPAO3	14.98	5.92

* qRT-PCR analysis of ATAF2 and selected candidate ATAF2 target genes at 5 hrs post leaf wounding vs non-wounded tissues. Fold changes are the average of two independent experiments. For each experiment leaf RNA was pooled from 5 individual test plants and qRT-PCR reactions were run in duplicate and normalized to 18 s rRNA levels.

Wild-type Columbia and ATAF2 knock-out (Salk_136355) plants were mechanically wounded 5 hr prior to RNA extraction.

### Identification of an ATAF2 consensus binding sequence

**Figure 9:**
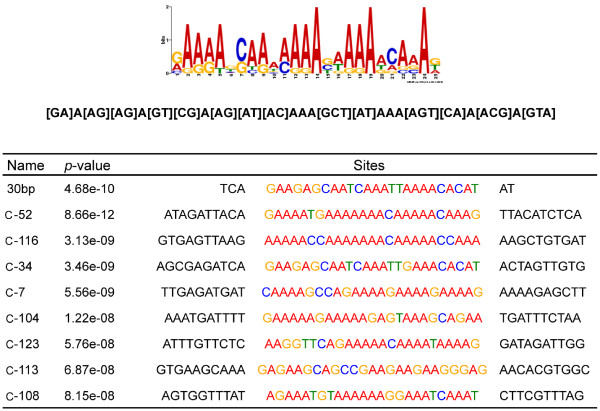
**Identification of the ATAF2 consensus binding sequence.** MEME analysis utilized the eight pull-down sequences listed. These sequences are all within 1000 bp upstream of a translational start site and were selected based on their ability to bind ATAF2 and / or display altered transcript accumulations of the corresponding mRNA in ATAF2 knockout plants. Brackets within the consensus sequence indicate the range of bases found at that position.

To expand upon the characterization of the ATAF2 binding sequence, pull-down sequences that were within 1000 bp of a translational start site and showed ATAF2 binding via EMSA and / or displayed altered transcript accumulations in ATAF2 KO plants were selected for motif analysis using the program MEME [21,22]. This selection criteria yielded eight different pull-down clones for which there was secondary evidence supporting ATAF2 binding. MEME analysis of these pull-down sequences revealed a consensus 25-nt sequence that corresponded to the 30-bp sequence identified above from the original C34 clone (Figure 9). The consensus sequence is predominantly A/T rich, but does contain repeating [CG]AAA motifs in forward or reverse orientations. Subsequently we expanded our analysis to 21 of the original clones identified by pull-down assay that were upstream and within 1000 bp of a known or predicted translational start site. MEME analysis of these genes also identified a similar A/T rich sequence within each of the additional clones (Table 1).

## Discussion

The induction of basal resistance responses can significantly impact virus accumulation and spread even within a susceptible host [23]. Thus, methods aimed at enhancing basal resistance could provide novel approaches for creating new forms of disease resistance. A number of studies indicate that SA mediates several anti-viral pathways that contribute to host basal defenses [23,24]. Recent studies by Lee et al., [25] suggest that during TMV infections SA-mediated signaling controls resistance mechanisms that involve the alternative oxidase pathway, RDR1 mediated RNA silencing systems, and other as yet uncharacterized defenses. These findings indicate that anti-viral defenses require the regulation of multiple host processes. Subsequently, viruses likely encode multiple countermeasures aimed at overcoming these defenses. The targeted degradation of ATAF2 by TMV suggests that this TF regulates host processes that affect the infection cycle. This possibility is supported by studies that show overexpression of ATAF2 leads to the induction of defense related genes and enhanced plant resistance to TMV [6]. In contrast, T-DNA knockout or transcription repressor plant lines show a marked decrease in the activation of these defense-associated genes. In particular, SA associated defense genes including *PR1* and *PR2* are reduced in transcriptional activation in the absence of ATAF2, either by knockout, transcriptional repression or TMV directed degradation [6]. These findings indicate that ATAF2 functions to enhance host basal defense processes and that its targeted degradation represents a potential TMV-directed counterdefense mechanism.

To better understand the ability of ATAF2 to regulate host gene expression we sought to characterize ATAF2’s function in the transcriptional regulation of cellular processes. The highly divergent C-terminal TAR regions of NAC proteins are thought to confer transcriptional activation of specific cellular functions including developmental and defense signaling pathways [19,26,27]. In yeast, the C-terminal TAR region of ATAF2 functioned to induce lacZ gene expression (Figure 4A), confirming ATAF2’s role as a transcriptional activator. Subsequently, a hexa-histidine-tagged ATAF2 protein readily functioned to pull-down Arabidopsis genomic DNA derived from the promoter regions of diverse genes. A representative sequence located within 1000 bp of the translational start site for the defensin-like protein At1G68907 was subsequently used to identify a 30-bp sequence that functioned as a *cis*-regulatory binding sequence for ATAF2. Most notably when co-expressed *in planta* with ATAF2 this 30-bp sequence directed the expression of a GUS reporter construct (Figure 4B). This 30-bp sequence also directed *in planta* GUS expression in response to a TMV infection (Figure 5). Thus, the induction of endogenous ATAF2 in response to infection is sufficient to drive gene expression from the identified ATAF2 binding sequence. Tissue wounding is also known to induce the transcription of ATAF2 [6,7]. Subsequent analysis of the transcript levels from nine of the original ATAF2 pull-down clones showed that eight were transcriptionally reduced by at least 2 fold in wounded tissues of the ATAF2 KO line in comparison to wounded tissues of the wild-type plant line (Table 2). The reduced transcriptional activation of these genes in the absence of ATAF2 indicates that ATAF2 contributes to the transcriptional regulation of these genes, further confirming the importance of the identified ATAF2 binding sequence within the promoters of these genes.

Wound induced activation of genes identified in the original ATAF2 pull-down assay indicated that these genes contain ATAF2 specific binding sequences. Motif analysis searches using the identified 30-bp binding sequence and promoter sequences shown to regulate gene expression upon the induction of ATAF2 or bind ATAF2 directly showed the presence of a 25-bp consensus sequence that was subsequently found in all analyzed pull-down sequences (Table 1). The identified 25-bp sequence is A/T rich and contains repeats of a [CG]AAA motif either consecutively or in reverse orientation. Previous studies have identified several NAC family DNA binding elements. In one study, *in vitro* selection was used to identify the DNA binding site of two functionally diverse NAC proteins: ANAC019, implicated in stress, and ANAC092, implicated in morphogenesis [16]. A core binding element of CGT[GA] was identified. This sequence is the reverse complement of the core binding sequence identified from three NAC proteins (ANAC019, ANAC055, and ANAC072) that recognizes a 63-bp sequence harboring CACG as the core DNA binding site [28]. Combined these findings suggest that diverse NAC proteins can bind similar sequences. However, other NAC proteins appear to bind different sequences. For example, NAC1 binds to a 21-bp DNA fragment containing an *as-1* element (TGACG) [19]. In wheat, a NAC protein binding consensus sequence was identified as [AG]G[AT]NNCGT[AG]NNNNN[CT]ACGT[AC]A[CT][CT] [29]. These previously identified NAC binding sequences including their core binding motifs are not present within the identified ATAF2 binding sequence, suggesting that ATAF2 recognizes a binding sequence different from that used by other NAC proteins. The A/T rich nature of the ATAF2 binding domain does have similarities to other plant based transcription binding domains. For example, the regulatory region of the pea plastoxyanin gene promoter is similarly A/T rich and is recognized by proteins containing high mobility group box domains that presumably modulate gene expression [30]. This diversity in sequence recognition reflects the large family of NAC proteins and the wide range of functions assigned to this TF family.

The 30 bp length of the DNA nuclease protected fragment and its nearly complete requirement for activity as determined by mutagenesis studies (Figure 6, 7) suggested ATAF2 functions as a multimer. This is consistent with previous studies that have shown several NAC proteins form and function as dimers [19,20]. In addition, crystallographic data for ANAC019 reveals an antiparallel β-sheet flanked by α-helices with a defined dimer interface that promotes both homo- and hetero-interactions along with a positively charged face that is thought to promote DNA binding [1,31]. To confirm the oligomeric status of ATAF2, purified ATAF2 NAC domain was examined through a size exclusion chromatography and a peak representing a dimeric form of the NAC domain was observed (Figure 8). Oligomerization of bacterial purified ATAF2 NAC domain suggests that ATAF2 likely functions as a dimer. Whether the functional ATAF2 oligomer is a homodimer or heterodimer formed with another NAC protein is unknown, but it is clear that the induction of ATAF2 in response to wounding or stress is required for the transcriptional activation of gene promoters encoding the identified binding sequence.

The role of ATAF2 in basal defense is not as yet resolved. However, it is interesting to note that several of the ATAF2 target genes identified in this study have links to defense responses. For example, Polyamine Oxidase 3 protein (PAO3) displays significant transcript reductions within ATAF2 KO tissues, indicating it is positively regulated by ATAF2 (Table 2). Furthermore, polyamines are known to accumulate in response to a number of environmental stresses including pathogen attack [32]. PAO3 functions within the peroxiosome, catalyzing accumulated polyamines and producing H_2_O_2_[33]. Uehara et al. [34] proposed the production of H_2_O_2_ from the catalysis of polyamines functions as a signal transducer for the activation of defense responses. Another example is the Oxidative Stress 3 protein (OXS3), which is also positively regulated by ATAF2. OXS3 is required for resistance to cadmium and co-localizes to the nucleus with the nucleosomal histone protein H4 where it is thought to function as a remodeling factor, moving the location of the nucleosome and altering gene expression [35]. Interestingly, cadmium treatment is linked to TMV resistance in plants and is correlated with the deposition of callose within the plasmodesmata and vascular tissues [36,37]. Ueki and Citovsky [38] identified a cadmium induced Glycine-Rich Protein (cdiGRP) that localizes to the vascular cell walls and promotes callose deposition. Overexpression of cdiGRP enhanced TMV resistance while its knockdown results in increased virus spread. In both of the above examples, ATAF2 directed regulation of PAO3 and OXS3 could enhance virus resistance via the production of H_2_O_2_ and the induction of cdiGRP, respectively. Such defense responses indicate that TMV’s targeted degradation of ATAF2 functions as an anti-defense countermeasure. However, confirming the role of ATAF2 in regulating these resistance pathways and their effect on mediating defense against TMV requires additional studies.

## Conclusion

We report here the identification of the ATAF2 binding sequence and its function in gene regulation in response to wounding and TMV infection. Identification of this binding sequence represents a significant step toward identifying the basal defense processes associated with ATAF2 expression as well as understanding the TMV counterdefenses targeting these processes.

## Methods

### Plant material, agroinfiltration, wounding, and virus inoculations

Plants were grown at 23°C under a 10 hour light / 14 hour dark cycle. Agroinfiltrations were done as previously described [39]. In summary, *Agrobacterium tumefaciens* strain GV3101 carrying the desired expression constructs were grown at 30°C overnight. Cultures were concentrated by centrifugation and resuspended in infiltration medium (10 mM MES, pH 5.7, 10 mM MgCl_2_, 150 μM acetosyringone) to an OD_600_ of 0.5 prior to leaf infiltration. Forty-eight hours post-agro-infiltration, the plant tissues were processed as described below. For wounding treatment, fully matured leaves of four-week old plants were wounded several times across the mid-vein using razor blades. Five hours after the wounding, the leaves were harvested and processed for analysis. For virus inoculations, TMV solutions of 0.1 to 0.5 mg/ml were rub-inoculated onto carborundum-dusted leaves.

### Plasmid constructs and protein expression

The full-length coding sequence of ATAF2 (AT5g08790) and its truncated version containing only the NAC domain (ATAF2-165, 1 aa to 165 aa) were each PCR-amplified to contain 5’ *Xho*I and 3’ *Kpn*I sites. The amplified fragments were ligated into the expression vector pTrcHisA (Life Technologies, Grand island, NY) to create pTrcHisA/ATAF2 and pTrcHisA/ATAF2-165 with N-terminus hexa-histidine tags. The ΔATAF2 deletion construct was created by PCR-amplification of ATAF2 fragments covering nucleotides 1 to 171 with 5’ *Xho*I and 3’ *Pst*I sites and nucleotides 376 to 852 with 5’ *Nsi*I and 3’ *Kpn*I sites. The two fragments were ligated together into *Xho*I and *Kpn*I cut pTrcHisA vector, to generate pTrcHisA/ΔATAF2. The purification of recombinant his-tagged ATAF2 and ΔATAF2 proteins was conducted as described previously [39]. Briefly, *Escherichia coli* BL21 (+) cells were grown at 37°C to an OD_600_ of 0.5 followed by pTrcHisA induction at 16°C with 1 mM isopropyl-1-thio-β-d-galactopyranoside. Bacterial cells were harvested and resuspended in lysis buffer containing 10 mM Tris, pH 8.0, 10% glycerol (v/v), 500 mM NaCl, and 10 mM imidazole. After sonication and centrifugation (17,000 g for 10 min), cell extracts were incubated with 1 ml (bed volume) of Ni-NTA affinity column (GE Healthcare, Piscataway, NJ) at 4°C for 1–2 h. The column was then washed with 10 column volumes of wash buffer (lysis buffer plus 10 mM imidazole). Proteins were eluted in buffer (lysis buffere plus 140 mM imidazole). Eluted proteins were analyzed by SDS–PAGE and protein concentration was determined via Bradford assay [40].

For the GUS reporter gene constructs, the −49 CaMV 35S minimal promoter and tandem repeats of 30-bp ATAF2 binding sequence were sequentially introduced into the pBI101 vector (Clonetech, Palo Alto, CA), upstream of the β-glucuronidase (GUS) coding sequence. In summary, two primers (5^′^TCGACCGCAAGACCCTTCCTCTATATAAGGAAGTTCATTTCATTTGGAGAGGAG−3^′^ and 5^′^GATCCTCCTCTCCAAATGAAATGAACTTCCTTATATAGAGGAAGGGTCTTGCGG-3^′^) covering the −49 to +1 region of the CaMV 35S promoter were inserted via *Sal*I and *BamH*I sites (underlined) into pBI101 to generate pBI/35SM-GUS. Primers carrying two copies of the 30-bp ATAF2 binding sequence and a *Xba*I restriction site (5^′^AGCTTGTCTAGAGATCAGAAGAGCAATCAAATTAAAACACATATTAGGATCAGAAGAGCAATCAAATTAAAACACATATTAGG−3^′^ and 5^′^TCGACCTAATATGTGTTTTAATTTGATTGCTCTTCTGATCCTAATATGTGTTTTAATTTGATTGCTCTTCTGATCTCTAGACA−3^′^) were then introduced into pBI/35Sm-GUS at the *Hind*III and *Sal*I sites (underlined) to create pBI/2X30bp-GUS. Similarly, two more copies of the 30-bp sequence **(**5^′^AGCTTGATCAGAAGAGCAATCAAATTAAAACACATATTAGGATCAGAAGAGCAATCAAATTAAAACACATATTAGT−3^′^ and 5^′^CTAGACTAATATGTGTTTTAATTTGATTGCTCTTCTGATCCTAATATGTGTTTTAATTTGATTGCTCTTCTGATCA−3^′^**)** were introduced into the *Hind*III and *Xba*I sites of pBI/2X30bp-GUS to generate pBI/4X30bp-GUS. All three GUS reporter gene constructs were transformed into *Agrobacterium tumefaciens* strain GV3101 [41].

### Size exclusion chromatography

The purified ATAF2 NAC domain (ATAF2-165) was incubated in buffer containing 25 mM Tris–HCl pH 7.5, 10% glycerol (v/v), 500 mM NaCl, and 0.5 mM EDTA for 20 min at room temperature. The incubated protein (~ 160 μg) was then run through a Superdex-200 HR 10/30 column (GE Healthcare, Piscataway, NJ) pre-equilibrated with the incubation buffer. Fractions (250 μl) were collected and a portion of each (50 μl) analyzed by SDS-PAGE, followed by Coomassie blue staining.

### Genomic pull-down assay

Plant genomic DNAs were extracted from four-week old Arabidopsis ecotype Shahdara leaf tissue using a standard CTAB genomic DNA isolation method [42]. Plant tissues were ground in CTAB buffer containing 2% Hexadecyl trimethyl-ammonium bromide, 100 mM Tris, pH 8.0, 20 mM EDTA, 1.4 M NaCl, and 0.2% β-mercaptoethanol. After incubation at 55°C for 1 hour, the CTAB/plant extract mixture was centrifuged at 12,000 g for 10 min. The supernatant was collected and the genomic DNA extracted with phenol/chloroform followed by ethanol precipitation.

Target sequences of ATAF2 were identified using a genomic pull-down assay described previously [18]. Briefly, Arabidopsis genomic DNA was digested with *EcoR*I and *Taq*I and ligated to two short linker sequences with corresponding restriction sites [18]. The DNA fragments were then incubated with recombinant his-tagged ATAF2 followed by precipitation with anti-polyHis antibody and protein A agarose (Life Technologies, Grand island, NY, Carlsbad, CA). After removing the bound protein with phenol/chloroform, the DNA fragments were PCR amplified and cloned into pCRII using TOPO TA Cloning Kit (Life Technologies, Grand island, NY).

### Electrophoretic mobility shift assays (EMSAs)

Double-stranded DNA probes were prepared either by PCR-amplification or by annealing complementary single-stranded DNA together. DNA probes were 5’ end-labeled with [γ-^32^P]ATP via T4 polynucleotide kinase (New England Biolabs, Ipswich, MA). For EMSA assays 100 to 300 fmol of each gel-purified labeled DNA fragment was mixed with various concentrations of purified ATAF2 protein in a 10 μl reaction containing 20 mM Tris–HCl, pH 8.0, 60 mM KCl, 5 mM MgCl_2_, 100 μg/ml BSA, 5% glycerol, 1 mM DTT, and 0.5 μg poly(dI-dC). Binding reactions were incubated at 25°C for 30 min, and then loaded onto a 5% (w/v) native polyacrylamide gel. Electrophoresis was carried out in 0.5X TBE buffer at 100 V for 2–3 h. Gels were vacuum-dried onto filter paper and visualized via PhosphorImager (Molecular Dynamics, Sunnyvale, CA). Quantitative analysis of DNA binding affinity of recombinant ATAF2 was performed on scanned gels using the ImageJ analysis tool.

### DNase I footprinting

5’ end-labeled DNA probe fragments were loaded on 5% non-denaturing polyacrylamide gels. After gel electrophoresis, probes were excised and recovered after diffusion overnight into 10 mM Tris–HCl, 1 mM EDTA, pH 8.0. The DNA-protein binding reaction was carried out as described above using purified ATAF2 protein (20 nM or 200 nM) and 10 fmol of probe DNA. This mixture was incubated for 30 min at 25°C followed by the addition of 50 μl of Ca/Mg solution (5 mM CaCl_2_ and 10 mM MgCl_2_). One minute later, 3 μl of RQ1 DNase, diluted at least 1:100 (determined empirically) from a 1 mg/ml stock, was added. RQ1 digestion was terminated after 1 min with 90 μl of 20 mM EGTA. Reactions were extracted with phenol:chloroform:isoamyl alcohol (25:24:1), and the DNA precipitated with ethanol and subjected to denaturing urea-polyacrylamide gel electrophoresis, followed by visualization using PhosphorImager (Molecular Dynamics, Sunnyvale, CA)

### GUS assays

GUS activity within plant tissue was visualized by histochemical staining with 5-bromo-4-chloro-3-indolyl-β-D-glucuronic acid (X-Gluc) as described previously [6]. For quantitative measurements of GUS activity, a modified fluorimetric *GUS* assay was used [43]. Briefly, plant tissues were ground in extraction buffer containing 150 mM sodium phosphate, pH 7.0, 10 mM EDTA, 10 mM β-mercaptoethanol, 0.1% Triton X-100, 0.1% sarcosyl, and 140 μM PMSF. After pelleting at 20,000 g for 15 mins, fluorometric substrate 4-methyl-umbelliferyl-β-D-glucuronide (4-MUG) was added to the supernatant to a final concentration of 1.0 mM. The mixed product was incubated in darkness at 37°C for 20 mins. 10 μl aliquots were then taken from each reaction and mixed with 190 μl stop buffer (0.2 M Na_2_CO_3_) in a black-wall clear-bottom 96-well plate. The fluorescent 4-Methylumbelliferone (MU) produced in the GUS reaction was measured using a SpectraMax M2 microplate reader (MTX Lab systems, Vienna, VA) with excitation at 365 nm and emission at 455 mm. A standard curve derived from six MU standards was included with every plate. Protein concentration in the extracts was determined by Bradford assay [40]. Final GUS activity was expressed as pmoles MU/min/mg protein. All experiments were repeated twice and the nonspecific GUS activity was normalized according to the relative GUS activity driven by the CaMV 35S minimal promoter.

### Real-time qRT-PCR

Leaf tissue from three to five individual test plants were pooled for RNA extraction. Total RNA was extracted using the RNeasy RNA extraction kit (Qiagen, Valencia, CA). 1 μg of total RNA was pre-treated with RQ1 DNase (Promega, Madison, WI) and used in a first strand cDNA synthesis reaction with SuperScript^TM^ II reverse transcriptase (Life Technologies, Grand island, NY). Real-time qRT-PCR reactions were performed using SYBR green PCR mix (Fermentas, Glen Burnie, MD) and an ABI Prism 7100 (Applied Biosystems, Foster City, CA) as previously described [6]. The 18 s rRNA was used as an internal control for normalization Primer sequences used for each of the selected genes are listed in Additional file 1: Table S1.

### Statistical analysis

Identification of statistically overrepresented motifs was done by using the motif search MEME program [22]. The 30-bp short ATAF2-binding sequence together with each selected candidate target genes identified from the genomic pull-down assay were chosen for the analysis**.** The motif search options were defined as “one occurrence per sequence” and motif width was set to be between 25 and 30 bp.

## Abbreviations

TFs: Transcription factors; NAC: NAM, ATAF1/2, CUC2; SA: Salicylic acid; TMV: *Tobacco mosaic virus*; EMSA: Electrophoretic mobility shift assay; KO line: Knockout line.

## Competing interests

The authors declare that they have no competing interests.

## Authors’ contributions

XW and JNC conceived, designed and wrote the manuscript. XW performed all experiments. Both authors read and approved the final manuscript.

## Supplementary Material

Additional file 1 **Table S1.** Primers used for real-time qRT-PCR. Click here for file
