# Supplementary material for: DNA binding specificity of ATAF2, a NAC domain transcription factor targeted for degradation by Tobacco mosaic virus
Source: BMC Plant Biol. 2012 Aug 31;12:157. doi: 10.1186/1471-2229-12-157 (PMC3507807; doi:10.1186/1471-2229-12-157)
Supplement: Additional file 1 — Table S1. Primers used for real-time qRT-PCR. [file 1471-2229-12-157-S1.docx]

Supplemental Table 1. Primers used for real-time qRT-PCR

|  | At5g08790 | forward | TGAGGATGCTTTGGAAGCTTTT |
| --- | --- | --- | --- |
|  | (ATAF2) | reverse | AGCGTCAGGCTGCAACAAC |
| C7 | At1g08540 | forward | CTTCTGGAGGAGCAACCTTC |
|  |  | reverse | TCCGAGCTTTCCTTTCAGTT |
| C32 | At2g28390 | forward | AGTCAAGGCAGGAAATCACC |
|  |  | reverse | TGTACAGCTGATGCAGACCA |
| C34 | At1g68907 | forward | GGACTGTAACTGCTTGCCTCT |
|  |  | reverse | TTATCTGTGCGGCAAGACTC |
| C52 | At3g26540 | forward | GCTCGCAGAGTATTTGACCA |
|  |  | reverse | ACTCATTGCATATCCCGACA |
| C104 | At3g62060 | forward | TTTCCCCAGAACTTGGTTTC |
|  |  | reverse | CTTGGATCTGCCAAGAGTCA |
| C108 | At4g19570 | forward | TGGGGGTCCAGTCATAAAAT |
|  |  | reverse | CTGAGGTTGTGGAAGAAGCA |
| C113 | At3g11700 | forward | TTAGAATCGGGTCACCACAA |
|  |  | reverse | ATCGAGGAGAGCTACGAGGA |
| C116 | At5g56550 | forward | TGGAGCAAAAAGAAAGTCATCT |
|  |  | reverse | GTTGGGTTTCTTGGAGATTGT |
| C123 | At3g59050 | forward | TTGCCTTTTCGCAACTACAG |
|  |  | reverse | TCCCCACCTTGATACCAAAT |
